# A Fluorescence Resonance Energy Transfer Aptasensor for Aflatoxin B1 Based on Ligand-Induced ssDNA Displacement

**DOI:** 10.3390/molecules28237889

**Published:** 2023-12-01

**Authors:** Kseniya V. Serebrennikova, Alexey V. Samokhvalov, Anatoly V. Zherdev, Boris B. Dzantiev

**Affiliations:** A.N. Bach Institute of Biochemistry, Research Center of Biotechnology of the Russian Academy of Sciences, Leninsky Prospect 33, Moscow 119071, Russia; ksenijasereb@mail.ru (K.V.S.); 03alexeysamohvalov09@gmail.com (A.V.S.); zherdev@inbi.ras.ru (A.V.Z.)

**Keywords:** mycotoxin, stem-loop aptamer, duplex DNA complex, ligand-induced displacement, FRET

## Abstract

In this study, a fluorescence resonance energy transfer (FRET)-based aptasensor for the detection of aflatoxin B1 (AFB1) was designed using a carboxyfluorescein (FAM)-labeled aptamer and short complementary DNA (cDNA) labeled with low molecular quencher RTQ1. The sensing principle was based on the detection of restored FAM-aptamer fluorescence due to the ligand-induced displacement of cDNA in the presence of AFB1, leading to the destruction of the aptamer/cDNA duplex and preventing the convergence of FAM and RTQ1 at the effective FRET distance. Under optimal sensing conditions, a linear correlation was obtained between the fluorescence intensity of the FAM-aptamer and the AFB1 concentration in the range of 2.5–208.3 ng/mL with the detection limit of the assay equal to 0.2 ng/mL. The assay time was 30 min. The proposed FRET aptasensor has been successfully validated by analyzing white wine and corn flour samples, with recovery ranging from 76.7% to 91.9% and 84.0% to 86.5%, respectively. This work demonstrates the possibilities of labeled cDNA as an effective and easily accessible tool for sensitive AFB1 detection. The homogeneous FRET aptasensor is an appropriate choice for contaminant screening in complex matrices.

## 1. Introduction

Infection of agricultural crops with molds before/after harvest, including those caused by improper storage, is a serious threat to the health of consumers [1]. The subsequent processing of raw materials, despite destroying the cells themselves, is not always able to remove the compounds they secrete in the process of life. In particular, a number of mold toxins (mycotoxins) are resistant to treatment [2,3]. The most widespread and persistent toxins are listed as substances controlled in food at the legislative level [4,5]. These toxins include a group of aflatoxins, the most important member of which is aflatoxin B1 (AFB1) [6,7]. The International Agency for Research on Cancer has also classified it as a class 1 carcinogen [8]. Agricultural products, such as maize, corn, nuts, etc., are most susceptible to AFB1 contamination. In this regard, the European Commission has set maximum residue limits for AFB1 in cereals and grain-containing products at 2 µg/kg [4]. The problem of food contamination with AFB1 is of significant economic importance due to the serious threat to human and livestock health, as well as the marketing of agricultural products. The improvement of existing analytical systems and the search for new cost-effective and highly sensitive ones is the task of paramount importance in solving this problem.

Currently, common methods used for AFB1 detection are liquid chromatography–mass spectrometry [9] and gas chromatography–tandem mass spectrometry (GC-MS) [10]; enzyme-linked immunosorbent assay (ELISA) [11] and immunochromatographic assay [12]. Each of these methods has its pros and cons. Chromatographic methods provide high accuracy and repeatability but are usually time-consuming and expensive due to their complex pretreatment processes, making them difficult to use for the high-throughput screening of a large number of samples. Although ELISA and immunochromatography are more user-friendly methods, they require multiple steps of time-consuming immobilization of reagents. The use of conventional antibodies provides good sensitivity and selectivity but is associated with problems of denaturation, batch-to-batch variability, and issues concerning chemical modification. Therefore, there is a need for a reliable, rapid and inexpensive method for the quantitative detection of AFB1.

In this regard, homogeneous fluorescent methods open up new possibilities due to their simplicity, speed, and sensitivity [13,14,15,16]. For example, fluorescence polarization/anisotropy-based immunoassays have been successfully used to detect AFB1 [17,18,19]. Moreover, homogeneous fluorescent assays can be easily implemented using the fluorescence resonance energy transfer (FRET) process, involving intermolecular non-radiative energy transfer between donor–acceptor pairs [20,21]. FRET-based assays provide real-time registration of an analytical signal and implement homogeneous interactions in solution without the need for immobilization of reagents, which greatly simplifies the testing.

Although antibodies dominate as recognition compounds in analytical systems, alternative receptors, in particular aptamers, are actively considered. Aptamers are single-stranded oligonucleotide receptor molecules that provide a number of advantages for biosensors due to their simple structure, low cost, efficient renaturation, the possibility of chemical synthesis, and the predictability of modification [22].

Like any oligonucleotides, aptamers are able to form duplexes with complementary strands. The possibility of displacement of a complementary chain in the presence of a ligand has been demonstrated. This phenomenon is called ligand-induced strand displacement. Tuning the length and localization of the complementary chain opens up opportunities for adjusting the sensitivity of the analysis [23,24].

Several sequences specific to AFB1 have been published [25,26,27]. Among them, the sequence with the repeating motif 5′-CGTGTTGTCTCTCTGTGTCTCG-3′ exhibits the highest affinity [27,28], which provides dissociation constants in the range from 10^−8^ to 10^−7^ M.

To date, several FRET aptasensors for the detection of AFB1 have been proposed using various aptamer/complementary DNA duplexes and donor–acceptor pairs. In these sensors, donor–acceptor pairs based on nanoparticles have been applied, including fluorescent polymer dots–Ag nanoparticles [29], CdZnTe quantum dots–Au nanoparticles [30], and ZnS quantum dots–Ag nanoparticles [25]. In addition, donor–acceptor pairs based on organic compounds, namely fluorescein–BHQ-1 [31], fluorescein–DABCYL [32], as well as binary systems of Cy5-BHQ-2 and Cy3-Cy5 fluorophores [33], have been implemented. More complex systems using a pair of FAM/BHQ-1 labeled ssDNA with non-overlapping complementary sites on the aptamer [34] or a Cy5-BHQ-2/Cy3-BHQ-2 pair [35] have also been characterized.

**Figure 1:**
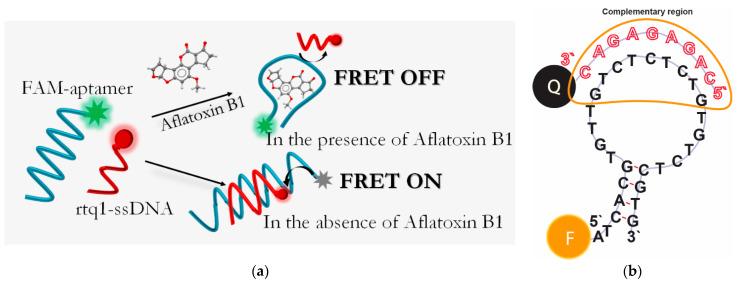
(**a**) Scheme of FRET-based aptasensor for AFB1 detection. (**b**) Representation of aptamer-cDNA binding site.

In this work, we reported an alternative FRET assay based on ligand-induced strand displacement for AFB1 detection using the low-molecular organic label RTQ-1 as an energy acceptor for carboxyfluorescein (FAM). The subject of development is a fluorescent molecular sensor representing molecular fluorescent probes (donor–acceptor pair) in solution, which, under the influence of the target analyte, generates a fluorescence signal as a result of preventing the FRET process. The sensing principle was based on the transition of complementary ssDNA labeled with RTQ-1 (RTQ1-cDNA) between bound and unbound states in the presence of AFB1. The transition was accompanied by the restoration of fluorescence of the FAM-labeled aptamer (FAM-Apt) under conditions when the distance for unbound RTQ1-cDNA exceeded the minimum distance required between RTQ1-cDNA and FAM-Apt for the FRET process to occur (Figure 1a). We have demonstrated that close proximity (within a few nucleobases) of the donor–acceptor pair for a short stem-loop aptamer is not a necessary condition to ensure the sensitive detection of the ligand of interest. The parameters of the assay, such as RTQ1-cDNA and FAM-Apt concentrations, as well as reaction conditions, have been optimized. The reached analytical parameters of AFB1 detection (linear range and detection limit) were determined. Finally, the applicability of the proposed FRET aptasensor was evaluated by analyzing white wine and corn flour samples spiked with AFB1 standards.

## 2. Results and Discussion

### 2.1. Principle of AFB1 Detection

**Figure 2:**
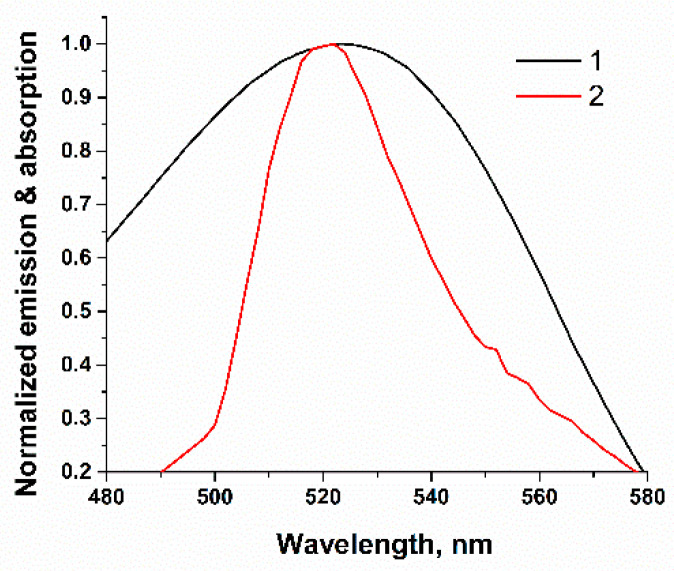
Overlap between absorption spectrum of RTQ1-cDNA (1) and the fluorescence emission of FAM-Apt (2).

A schematic illustration of the FRET-based aptasensor is demonstrated in Figure 1. In this assay, a specific FAM-labeled truncated 26-mer aptamer (5′-FAM-ATCACGTGTTGTCTCTCTGTGTCTCGTG-3′) with a stem-loop secondary structure stabilized by four base pairs was used. To detect AFB1, ssDNA complementary to either the 5′ or 3′ end of the aptamer was typically used to ensure the proximity between the quencher and the fluorophore [32,33,34,35,36]. Considering the flexibility of ssDNAs [37], we applied an ssDNA complementary to the loop region of the aptamer (Figure 1b) identified in previous studies as the binding site for AFB1 [28]. This choice provides the potential possibility of directly affecting the aptamer binding site rather than its structural integrity in the event of disruption of the terminal region of the ssDNA stem. Therefore, a short 9-mer ssDNA labeled with a quencher (RTQ1) at the 3′ end (5′-CAGAGAGAC-RTQ-1-3′) was complementary to the sequence in the loop region (Figure 1b) and close to the 5′ end of the aptamer. The length was chosen based on our previous study, showing that the formation of 23 H-bonds between that of the aptamer and ssDNA under high salt conditions (10 mM Mg^2+^, 10 mM Ca^2+^ and 100 mM Na^+^) is enough to have a dissociation constant close to 10^−8^. Under similar salt conditions, the dissociation constant of apamer-AFB1 was found to be 49 ± 2 [28]. Therefore, the interaction constants of aptamer–ligand and aptamer–ssDNA would be comparable [38]. In the absence of AFB1, the aptamer hybridized with the cDNA, as a result of which the RTQ1 turned out to be in close proximity to FAM, and the fluorescence of the latter was quenched. The spectral overlap shown in Figure 2 confirms the possibility of the FRET process for this donor–acceptor pair. In the presence of AFB1, a structural switch of the aptamer occurred with the formation of an analyte/aptamer complex, as a result of which cDNA was dehybridized from the aptamer and the fluorescence of FAM was recovered.

### 2.2. Optimization of Assay Conditions

**Figure 3:**
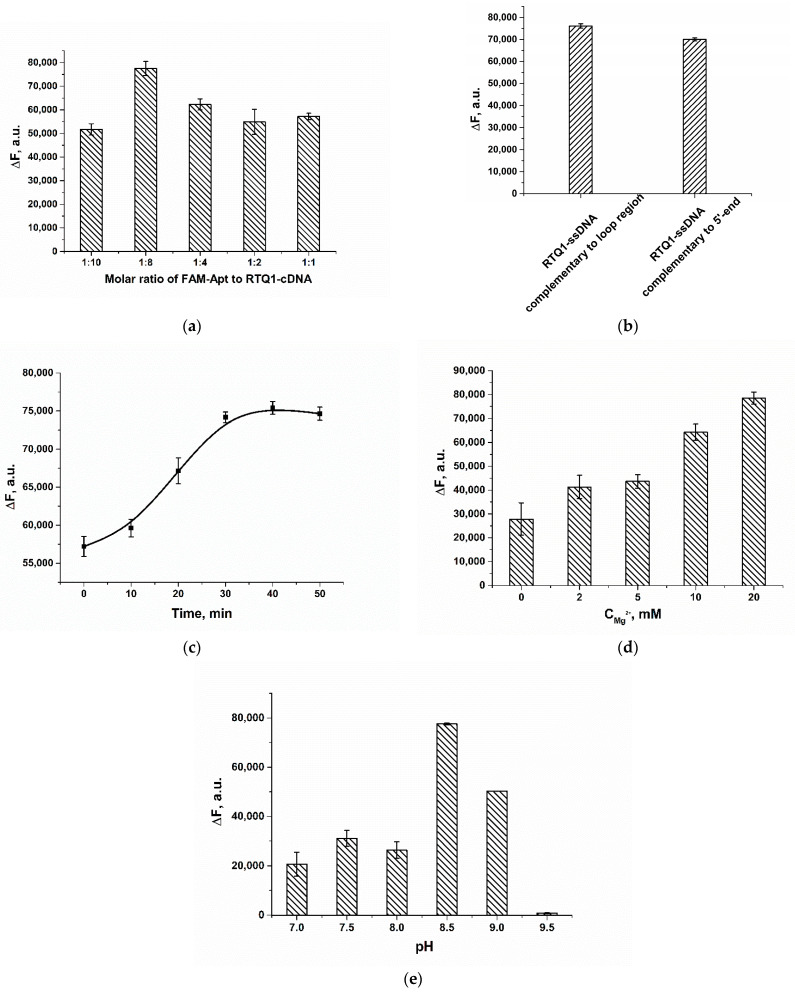
Optimization of assay parameters, given in terms of the difference (∆F) between fluorescence intensity in the presence of 200 nM AFB1 and in the absence of analyte (blank). (**a**) Variation in FAM-Apt: RTQ1–cDNA molar ratio. (**b**) The recovered fluorescence of FAM-Apt in the presence of 200 nM AFB1 obtained using ssDNAs complementary to different regions of Apt. (**c**) The recovered fluorescence of FAM-Apt in the presence of 200 nM AFB1 obtained after different incubation time. (**d**) Effect of different concentrations of Mg-acetate in WB on the recovered fluorescence. (**e**) Effect of buffer pH on the recovered fluorescence of FAM-Apt. The error bars represent the standard deviation of the triplicate measurements.

To optimize the sensing performance, the ratios of the RTQ1-cDNA and FAM-Apt, the time of assay, the concentration of Mg^2+^ and the pH of the working buffer (WB) were varied. As shown in Figure 3a, as the molar ratio increases, the recovered fluorescence of FAM-Apt in the presence of the analyte gradually increases. However, the opposite effect is observed at a molar ratio of 1:10, which could be explained by the high stability of the FAM-Apt/ RTQ1-cDNA complex and a decrease in the binding of the aptamer to the analyte. Therefore, for further experiments, a molar ratio of 1:8 was chosen, which provides the maximum recovered fluorescence, whereas the concentrations of FAM-Apt and RTQ1-cDNA were 5 and 40 nM, respectively.

The recovered fluorescence of FAM-Apt using ssDNA complementary to the 5′ end of the aptamer (5′-ACA ACA CGT G-(RTQ1)-3′) was also studied to compare the quenching effectiveness between the usually used quencher-labeled-cDNA to the terminal region and the chosen cDNA to the loop region. The terminal cDNA–FAM-Apt ratio was optimized, and a ratio of 4:1 was selected. As shown in Figure 3b, both RTQ1-labeled cDNAs to the 5′ end and loop of the aptamer provide the equal change in FAM fluorescence in the presence of 200 nM of AFB1, thereby no significant decrease in FRET efficiency using cDNA complementary to the loop region was observed.

The time for the target-induced fluorescence recovery was also investigated. As can be seen in Figure 3c, fluorescence was restored for up to 30 min, after which the ∆F within an error remained constant. In this regard, fluorescence was measured 30 min after the reaction was initiated by adding AFB1 to the mixture of the FAM-Apt and RTQ1-cDNA.

The absence of natrium ions influence on the aptamer–AFB1 interaction was pinpointed previously [31]. Therefore, the next key parameters affecting the formation of the aptamer/cDNA duplex and the binding of the aptamer to the analyte, were the concentration of Mg^2+^ and buffer pH. Increasing the magnesium acetate concentration in the range of 1–20 mM caused a gradual increase in the restored fluorescence (Figure 3d). Here, the optimal Mg^2+^ concentration in WB was determined to be 20 mM. Previously published studies also showed the effectiveness of using Mg^2+^ at a concentration of 20 mM [31,39]. Finally, the restored fluorescence was tested in buffers over the pH range of 7–9 to determine the optimum acidity. As shown in Figure 3e, the maximum response for 200 nM AFB1 was observed at pH 8.5. The low fluorescence at acidic and neutral conditions is consistent with the pH optimum of fluorescein fluorescence. Under strong alkali conditions, the signal is absent, which is governed by intermolecular interaction in the aptamer-ligand–cDNA system. Thus, the FAM-Apt/RTQ1-cDNA molar ratio of 1:8, the reaction time of 30 min, and a WB containing 20 mM of Mg^2+^, pH 8.5 were determined to be optimal.

### 2.3. Aflatoxin B1 Detection Performance

**Figure 4:**
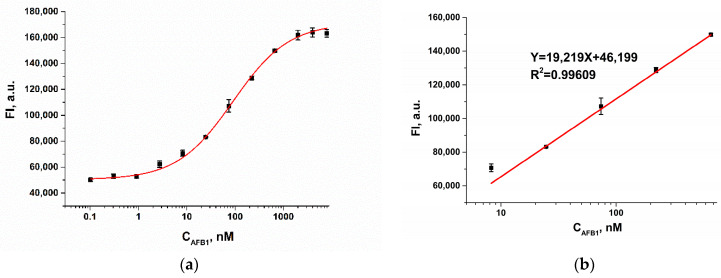
(**a**) Calibration curve for AFB1 (*n* = 3) obtained in WB. (**b**) Linearity of fluorescence intensity with respect to AFB1 concentrations.

A quantitative fluorescence assay of AFB1 was performed under optimized conditions. As shown in Figure 4a, the fluorescence intensity increased with increasing AFB1 concentration and reached a plateau when the analyte concentration exceeded 1000 nM. The dependence of the fluorescence intensity on the AFB1 concentration was described through the use of the following equation:

FI=175,465+50,022−175,465(1+(C103)0.7

where FI is the fluorescence intensity of FAM-Apt, a.u.; C is AFB1 concentration, nM. The limit of detection, calculated as three times the standard deviation of the fluorescence intensity of the blank sample, was 0.7 nM (0.2 ng/mL).

Since the accuracy of the analysis decreases at the upper and lower plateaus, we limited the use of the sigmoid dependence to the evaluation of the dynamic range. The dynamic range (Figure 4b) varied from 4.8 to 588.2 nM and was described through the use of the following linear equation: FI=19,219∗C+46,199 (FI is the fluorescence intensity of FAM-Apt, a.u.; C is AFB1 concentration, nM) with a correlation coefficient of 0.996.

### 2.4. Selectivity of the FRET-Based Aptasensor in AFB1 Detection

**Figure 5:**
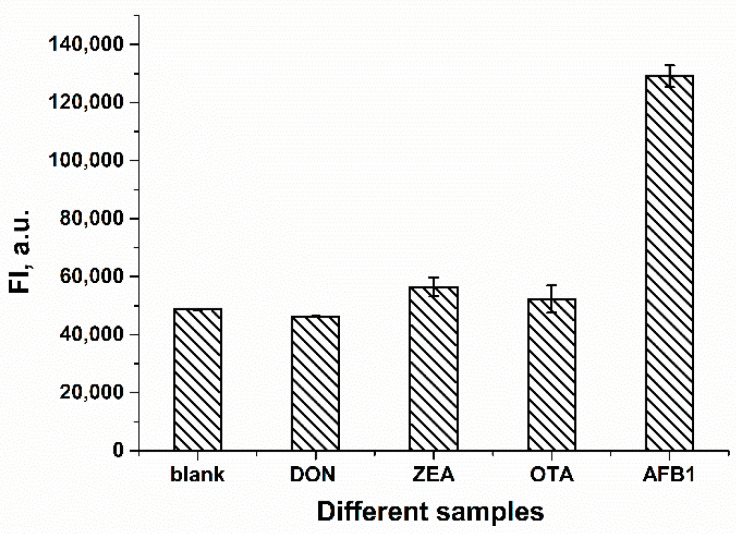
Fluorescence intensities in the absence (blank) and presence of various mycotoxins (*n* = 3). The concentration of AFB1 was 200 nM, and the concentration of other mycotoxins was 1 µM.

To evaluate the selectivity of the proposed FRET-based aptasensor for AFB1, several other mycotoxins were also tested. For the experiment, 62.4 ng/mL (200 nM) AFB1 and an excess of other mycotoxins amounting to 403.8 ng/mL OTA, 318.4 ng/mL ZEA and 296.3 ng/mL of DON were added to the mixture of FAM-Apt and RTQ1-cDNA, respectively. Molar concentrations of mycotoxins for selectivity testing were 200 nM for AFB1 and 1 µM for other interfering mycotoxins. The excess of the non-specific concentration of toxins was chosen to emphasize the lack of their effect on fluorescence intensity. As shown in Figure 5, the fluorescence intensity of other mycotoxins did not change compared to the blank. At the same time, the addition of AFB1 led to a significant restoration in terms of the fluorescence of FAM-Apt. The results confirm that the FRET-based aptasensor has a high selectivity for AFB1.

### 2.5. Detection of AFB1 in Food Samples

To assess the practical applicability and reliability of the developed FRET-based aptasensor, AFB1 spiked samples of food matrixes were analyzed. Since the extraction of contaminated corn flour was carried out using methanol, this extractant formed the basis of the final extract. To avoid solvent interference, samples were diluted before testing. As for the wine samples, they were diluted with a buffer since the wine initially contained alcohol in its composition. This simple preparation allowed us to minimize the influence of the sample on the analysis result. In addition, a calibration curve in the buffer was used for the fortified sample analysis.

As shown in Table 1, the recovery of AFB1 in white wine ranged from 76.7 to 91.9%, whereas the AFB1 recovery in corn flour samples ranged from 84.0 to 86.5%. These results indicate the feasibility of the developed aptasensor for the detection of aflatoxin B1 in food samples.

### 2.6. Comparison of the Developed FRET Aptasensor with Other Methods

**Figure 6:**
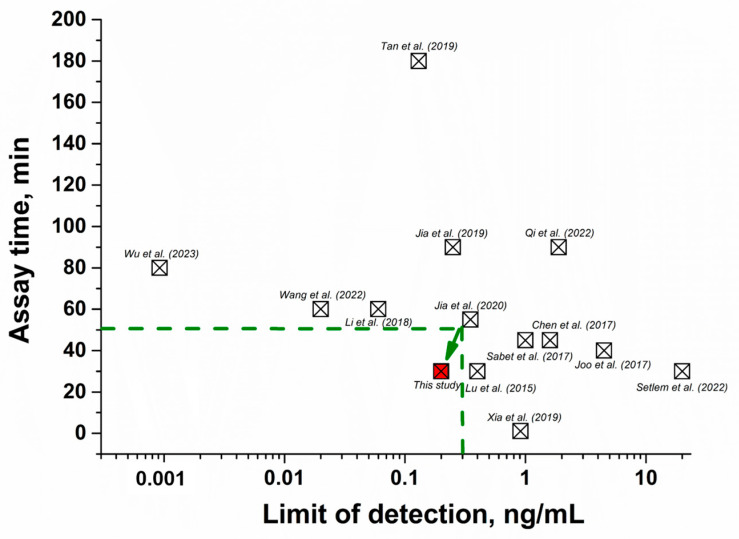
Comparison of FRET aptasensors for AFB1 (listed in Table 2) in terms of detection limit and analysis time [31,34,40,41,42,43,44,45,46,47,48,49,50]. The red square highlights the data obtained in this study. The green dotted line indicates the area of optimal characteristics of FRET aptasensors (rapidity and sensitivity of the analysis), where the detection limit of mycotoxin is up to 0.3 ng/mL, and the assay duration is up to 50 min.

The performance of the given aptasensor was compared with other FRET-based assays in terms of linear range, detection limit, and time. As shown in Table 2, previously proposed FRET aptasensors are characterized either by high sensitivity but a long time required to perform the assay [31,34,40,41,42,43,44] or rapid testing with low sensitivity [45,46,47,48,49,50]. Thus, by comparing existing FRET aptasensors in terms of sensitivity and analysis time (Figure 6), our aptasensor demonstrated the optimal combination of these parameters and allowed us to consider its development as a potential tool for the determination of AFB1 in food.

## 3. Conclusions

In summary, a homogeneous fluorescent method for the determination of aflatoxin B1 was developed by designing a donor–acceptor pair of a truncated AFB1-specific aptamer labeled with FAM and RTQ1-labeled ssDNA complementary to the loop region of the aptamer to implement an analysis based on the FRET process. This FRET-based aptasensor has the following obvious advantages: (1) the aptasensor allows one-step detection of aflatoxin B1; (2) the time required for analysis is short being equal to 30 min; (3) high sensitivity and specificity of the aptasensor; (4) applicability to different food samples. Thus, the excellent performance of the developed FRET-based aptasensor makes it an effective method for the simple determination of aflatoxin B1.

## 4. Materials and Methods

### 4.1. Reagents and Materials

The standard solutions of aflatoxin B1 (AFB1), ochratoxin A (OTA), zearalenone (ZEA), and deoxynivalenol (DON) were obtained from Chimmed (Moscow, Russia). AFB1 aptamer 5′-(FAM)-AT CAC GTG TTG TCT CTC TGT GTC TCG TG-3′, as well as its complementary ssDNAs 5′-CAG AGA GAC-(RTQ1)-3′ and 5′-ACA ACA CGT G-(RTQ1)-3′ were custom-synthesized and purified by Syntol (Moscow, Russia). Real-Time Quencher-1 (RTQ1) is a low molecular organic quencher produced by Syntol (Moscow, Russia) with λ_max_(abs) = 520 nm and a quenching operating range of 470–570 nm, with the absorption of FAM-fluorescence being 2 times better than BHQ1 [51,52]. Tris(hydroxymethyl)aminomethane, magnesium acetate, polyvinylpyrrolidone (PVP-10) and sodium acetate were obtained from Sigma Aldrich (St. Louis, MO, USA). Amicon Ultra-15 centrifuge filter units (3 kDa cutoff) were purchased from Merck Millipore (Carrigtwohill, Ireland). All reagents applied in experiments were of analytical grade.

All aqueous solutions were prepared in ultrapure water obtained via a Simplicity Milli-Q^®^ system from Millipore (Burlington, MA, USA). All interactions were carried out in a working buffer (WB; Tris-acetate containing 20 mM Mg-acetate and 100 mM Na-acetate, pH 8.5) at 25 °C. Stock solutions of the aptamer and oligonucleotide were prepared by dissolving lyophilized DNA in deionized water to the concentration of 200 µM. Fluorescence measurements were performed in black 96-well plates (NUNC Maxisorp) obtained from Thermo Fisher Scientific (Waltham, MD, USA). A corn flour negative sample was provided by Trilogy Reference Material (Washington, DC, USA). White wine was purchased from the local market.

### 4.2. Apparatus

Fluorescence intensity was measured using the multi-mode microplate reader CLARIOstar Plus (BMG Labtech, Ortenberg, Germany) in the “fluorescence intensity” or “kinetic slow” mode using an excitation filter (482 ± 16 nm), dichroic mirror (504 nm), and emission filter (520 ± 10 nm) with an automatic adjustable focal length. Aptamer and oligonucleotide concentrations were verified using a NanoDrop2000 microvolume spectrophotometer by examining the optical density at 260 nM (Thermo Scientific, Waltham, MA, USA). All experiments were carried out under the same constant temperature—25 °C.

### 4.3. Optimization of Assay Conditions

To determine the optimal concentration of RTQ1-cDNA, a series of RTQ1-cDNA dilutions in WB from a concentration of 50 nM with a dilution step of 2 were mixed with 5 nM FAM-Apt in the presence and absence of 200 nM AFB1.

To obtain the optimal reaction time, 5 nM FAM-Apt was mixed with 40 nM RTQ1-cDNA in the presence and absence of 200 nM AFB1, followed by measurement of fluorescence intensity in kinetic mode for 50 min after gentle agitation of the plate for 5 min.

The optimal concentration of Mg^2+^ was established by mixing 5 nM FAM-Apt and 40 nM RTQ1-cDNA with WB, containing Mg^2+^ in the concentration range from 2 mM to 20 mM in the presence and absence of 200 nM AFB1.

The optimal pH was determined via the fluorescence measurements of the FAM-Apt/RTQ1-cDNA complex in WB with pH varied in the range of 7–9 in the presence and absence of 200 nM AFB1.

Optimization data are presented as a dependence of ∆F = FI_200nM_ − FI_blank_ on the parameter under study, where FI_200nM_ is the intensity value in the presence of 200 nM AFB1, and FI_blank_ is the fluorescence intensity in the absence of the analyte. The values of the varied parameters under which the restored fluorescence (∆F) reached its maxima were chosen as optimal ones.

### 4.4. Aflatoxin B1 Detection with FRET-Based Aptasensor

For competitive detection of AFB1, 100 µL of AFB1 standards were added to the microplate wells in the concentration range from 1 µM to 0.1 nM. Then, 50 μL aliquots of FAM-Apt and RTQ1-cDNA were added, followed by 30 min incubation at room temperature. Before measuring fluorescence, the microplate was stirred for 30 s with the CLARIOstar option of the reader to ensure equilibrium. The analysis was carried out in triplicate. Calibration curves were obtained by plotting the fluorescence intensity of the FAM-Apt versus the logarithm of AFB1 concentration using Origin 9.0 software (OriginLab Corp., Northampton, MA, USA). The limit of detection was calculated as the concentration according to the triple standard deviation above the blank mean.

### 4.5. Selectivity Testing

The selectivity of the developed FRET-based aptasensor was evaluated using the following mycotoxins: ochratoxin A (OTA), zearalenone (ZEA), and deoxynivalenol (DON). Mycotoxins were diluted in WB to a final concentration of 1 µM and added to microplate wells with the following possibility to interact with the added FAM-Apt and RTQ1-cDNA as described in Section 4.4.

### 4.6. Sample Preparation

Before the pretreatment of samples, wine and corn flour were spiked with different concentrations of AFB1, taking into account further dilution of food samples. White wine was processed according to a previously described protocol [53,54]. Briefly, 0.02 g/mL PVP-10 was added to an aliquot of wine to decolorize the sample. After 5 min of stirring, the sample was filtered on Amicon Ultra-15 centrifuge filter units for 30 min at 15,000× *g*. After adjusting the pH to 8.5 with 1 M potassium hydroxide, magnesium acetate was added to the final concentration of 20. To remove the precipitate, the sample was centrifuged at 4000× *g* for 15 min.

The corn flour was pretreated according to the protocol [55,56]. Briefly, corn flour was extracted with a mixture of methanol and water in a ratio of 30:70. The resulting mixture was filtered through 0.45 μm membrane filters and centrifuged for 15 min at 2500× *g* to remove the precipitate.

Wine and corn extracts were diluted with WB 10 and 25 times, respectively, prior to their testing.

## Figures and Tables

**Table 1:** Detection of AFB1 in white wine and corn flour (*n* = 3).

Samples	Added *, ng/g	Found, ng/g	Recovery, %
**Wine**	3	2.3 ± 0.5	76.7
	8	6.4 ± 1.5	80.0
	26	23.9 ± 4.6	91.9
**Corn**	7.5	6.3 ± 2.5	84.0
	20	17.3 ± 2.5	86.5
	65	55.3 ± 1.8	85.1

* The spiked wine was diluted 10 times; the spiked corn flour was diluted 25 times.

**Table 2:** Comparison of the analytical performance of the FRET-based aptasensors for AFB1 detection.

Sensor Components	Linear Range	Limit of Detection	Assay Time	Sample/ Dilution	Minimum Detectable Concentration	Reference
FAM-Apt/RTQ1-cDNA	2.5–208.3 ng/mL	0.2 ng/mL	30 min	Wine and corn flour/10 and 25 times	7.5 ng/g for corn and 3 ng/g for wine	This study
**A fluorescein amidite (FAM)-labeled AFB1-specific aptamer/graphene oxide**	4.5–300 ng/mL	4.5 ng/mL	40 min	Rice seeds/no dilution	4.5 ng/mL	[45]
**AFB1 aptamer modified with quaternized tetraphenylethene salt/graphene oxide**	0–3 ng/mL	0.25 ng/mL	90 min	milk, corn and rice/3 times	1.92 ng/mL	[40]
**Aptamers-modified mesoporous silica nanoparticles loaded with Rh6G**	0.5–50 ng/mL	0.13 ng/mL	3 h	Corn oil, corn/not mentioned	1 ng/g	[41]
**FAM-labeled AFB1 aptamer/cDNA modified with carboxytetramethylrhodamine**	5–100 ng/mL	1.6 ng/mL	45 min	Infant rice cereal samples/5 times	5 ng/mL	[46]
**Label-free aptamer/fluorescein-labeled complementary strand/quencher (BHQ1)-labeled complementary strand**	0.02–1000 ng/mL	0.02 ng/mL	60 min	Beer and corn flour/20 times	Not mentioned	[34]
**TAMRA-labeled aptamer/metal-organic frameworks UiO-66-NH_2_**	0–180 ng/mL	0.35 ng/mL	55 min	milk, corn and rice powder/3 times	1.52 ng/mL	[42]
**Aptamer modified with CdTe quantum dots/graphene oxide**	0.5 ng/mL to 50 µg/mL	0.4 ng/mL	30 min	Peanut oil/no dilution	0.5 ng/mL	[47]
**Aptamer-conjugated quantum dots adsorbed to Au nanoparticles**	3–125 ng/mL	1 ng/mL	45 min	Peanut and rice/not mentioned	≈1.6 ng/mL	[48]
**Two FAM-labeled aptamer/two black hole quencher-labeled anti-aptamer**	1–200 ng/mL	0.91 ng/mL	1 min	Peanut oil and broad bean paste/no dilution	50 ng/mL	[49]
**FAM-labeled aptamer/BHQ1-labeled cDNA**	0.06–156 ng/mL	0.06 ng/mL	1 h	Wine and maize flour/100 times	0.2 ng/g for wine and 0.4 ng/g for maize	[31]
**Alexa Fluor 488 labeled aptamer/graphene oxide sheets**	0.2–200 ng/mL	20 ng/mL	30 min	Groundnut/no dilution	20 ng/mL	[50]
**dual-AFB1 aptamers/Cas12a-crRNA/ssDNA-FAM/MXenes-Ti3C2Tx**	0.001–80 ng/mL	0.92 pg/mL	80 min	Peanut/no dilution	1 ng/mL	[43]
**SYBR Gold/aptamer/single-walled carbon nanohorns**	5–200 ng/mL	1.89 ng/mL	1.5 h	Soybean sauce/100 times	10 ng/mL	[44]

## Data Availability

The data that support the findings of this study are available from the corresponding author upon request.
